# Data of patients undergoing rehabilitation programs

**DOI:** 10.1016/j.dib.2020.105419

**Published:** 2020-03-16

**Authors:** Ruggiero Seccia, Marco Boresta, Federico Fusco, Edoardo Tronci, Emanuele Di Gemma, Laura Palagi, Massimiliano Mangone, Francesco Agostini, Andrea Bernetti, Valter Santilli, Carlo Damiani, Michela Goffredo, Marco Franceschini

**Affiliations:** a DIAG - Sapienza University of Rome, Via Ariosto 25, Rome 00185, Italy; b Department of Computer, Control and Management Engineering Antonio Ruberti, Sapienza University of Rome, Italy; c Department of Anatomy, Histology, Forensic Medicine and Locomotor Sciences School of Pharmacy and Medicine, Sapienza University of Rome, Italy; d Neurorehabilitation, IRCCS San Raffaele Pisana, Rome, Italy

**Keywords:** Rehabilitation data, Personalized healthcare, Barthel index

## Abstract

In this data article, we present a dataset made up of personal, social and clinical records related to patients undergoing a rehabilitation program. Data refers to records registered in the “Acceptance/Discharge Report for the rehabilitation area” (ADR) which implements the Italian law (DGR 731/2005) and refer to hospitalization at the rehabilitation hospital of Rome “San Raffaele” in the years from 2015 to 2018 of patients suffering from orthopedic and neurological pathologies. For each ADR report, the clinical status of the patient at the date of acceptance and discharge is reported using, among other, the Barthel index as a measure of the Activities Daily Living of the patient.

These data can be used to understand the influence of many different factors in the rehabilitation progress of clinical patients.

Specifications table

**Table:** 

Subject Area	Medicine and Dentistry
More specific subject area	Orthopedics, Sports Medicine and Rehabilitation
Type of data	Spread-sheet tables
How data was acquired	Acceptance/Discharge report (ADR) Spreadsheet
Data format	Raw and filtered
Parameters for data collection	Data were collected from the Acceptance/Discharge reports (ADR) of patients entering a rehabilitation program in the years 2015–2018
Description of data collection	In accordance with the Code of Ethics of the World Medical Association for each patient the Acceptance/Discharge report (ADR) is coded. The dataset shared for analysis was anonymized to respect privacy compliance.
Data source location	San Raffaele rehabilitation hospital, Rome, Italy
Data accessibility	Repository name: Figshare
	Data identification number:
	10.6084/m9.figshare.11663277
	Direct URL to data: https://figshare.com/s/7aab7ad2d351f53dbe2f

## Value of the Data

• Data can be used to understand the role of different factors in the rehabilitation course of patients with orthopedic or neurological pathologies
• Statistical and data-driven techniques can be employed to extrapolate patterns and trends in the data
• Data can be used by machine learning practitioners for benchmarking and testing the performance of Machine Learning algorithms

## 1 Data description

We present a dataset made up of personal, social and clinical records related to patients entering a rehabilitation program. Each patient has further associated a performance indicator called Barthel Index (BI) - which is widely recognized as a metric for assessing the Activities Daily Living (ADL) level. For each patient, the BI is reported both when entering and leaving the hospital. A description of the BI can be found in Section 1.1.

It is of key importance to assess in advance the potential functional improvement of patients undergoing a rehabilitation program because this may help in developing precision medicine tools and to assess a rehabilitative path that is patient specific (see e.g. [3]). The underlying idea is that the patient’s response to a therapeutic treatment depends both on the general clinical status and also on some other side conditions that overall interact with each other in a highly nonlinear way. The dataset can be used within a data-driven toolbox for predicting the BI in discharge using all information deriving from the status of the patient when entering the program. Advanced data-driven methods such as Deep Neural Networks [4] can be applied to extract patterns and predictions out of this data, see for an introduction to the topic and algorithms for training these models [2], [6], [7].

Data have been extracted from records registered in the “Acceptance/Discharge Report for the rehabilitation area (ADR) which implements the Italian law (DGR 731/2005) and refer to hospitalization at the rehabilitation hospital of Rome “San Raffaele” in the years from 2015 to 2018 of patients suffering of orthopedic and neurological primary pathologies. We refer for short to these two groups of patients as orthopedic and neurological patients. Data have been treated in accordance with The Code of Ethics of the World Medical Association (Declaration of Helsinki [9]). Consent was obtained for experimentation with human subjects. All the datasets were anonymized to respect privacy compliance. Within the ADR, the pathologies are reported following the standard International Classification of Diseases, Ninth Revision, Clinical Modification (ICD9-CM) [1].

The following files are made available and will be described in detail in the following sections.

- **Dataset.csv**: Raw dataset containing information about each patient;
- **Cleaned_ Dataset.csv**: dataset obtained after the preprocessing procedure described in section 2.2;
- **Orthopedic.csv**: cleaned dataset which refers only to orthopedic patients;
- **Neurologial.csv**: cleaned dataset with only neurological patients;
- **Encoding Features.xlsx**: files divided in three sheets each containing the table to assign the codes ICD9-CM to the corresponding clinical categories as described respectively in Table 4, Table 5, Table 6.

Moreover, Table 1 reports the Barthel index scores for each daily life activity. Table 2 contains the list of the features of the raw dataset and a brief description of these features. Table 3 reports the encoding of the social fields used in the cleaned Dataset. Table 4, Table 5, Table 6 report the codification of the ICD9-CM codes corresponding to COD_1 (the pathology responsible for the rehabilitation intervention), COD_2 (main pathology), COD_3-10 (associated pathologies or comorbidities). Table 7 reports as a matter of example the list of values for one of the impairments (the manipulation impairment, corresponding to COD_15). Finally, Table 8 reports a summary of the Datasets reported in the four files described below.

**Table 1:** Barthel Index score per daily life activity

Activity	Scores				
	Unable to perform task	Attempts task but unsafe	Moderate help required	Minimal help required	Fully independent
Personal Hygiene	0	1	3	4	5
Bathing	0	1	3	4	5
Feeding	0	2	5	8	10
Toilet	0	2	5	8	10
Stair climbing	0	2	5	8	10
Dressing	0	2	5	8	10
Bowel control	0	2	5	8	10
Bladder control	0	2	5	8	10
Ambulation	0	3	8	12	15
Wheelchair	0	1	3	4	5
Chair/bed transfers	0	3	8	12	15

**Table 2:** Description of the features in the starting dataset.

Name	ADR Code	Type of feature	Potential values
Sex	Sex	Binary	[0 (F), 1 (M)]
Age	Age	Integer	[18–97]
Hospitalization days	GGP	Integer	[0–295]
Marital status	MaritalStatus	Integer	[1–8]+99
Education	LevelOfEducation	Integer	[1–5]+99
Pathology responsible for the rehabilitation intervention	COD_1	Categorical	ICD9-CM
Main pathology	COD_2	Categorical	ICD9-CM
Associated pathology or comorbidities	COD_3-10	Categorical	ICD9-CM +99
Cognitive impairment	COD_11	Integer	[1–8]+999
Behavior impairment	COD_12	Integer	[1–8]+999
Communication/language impairment	COD_13	Integer	[1–7]+999
Sensory impairment	COD_14	Integer	[1–8]+999
Manipulation impairment	COD_15	Integer	[1–7]+999
Balance impairment	COD_16	Integer	[1–7]+999
Locomotion impairment	COD_17	Integer	[1–10]+999
Cardiovascular impairment	COD_18	Integer	[1–8]+999
Respiratory system impairment	COD_19	Integer	[1–8]+999
Kind of ulcer	COD_20	Integer	[1–7]
Sphincter control impairment	COD_21	Integer	[1–5]+999
Urinary system impairment	COD_22	Integer	[1–7]+999
Nutrition impairment	COD_23	Integer	[1–7]
Post-Comatose patient	COD_24	Integer	[1–3]
Barthel Index at admission	COD_25	Integer	[0–100]
Feeding at admission	COD_25_1	Integer	[0,2,5,8,10]
Bathing at admission	COD_25_2	Integer	[0,1,3,4,5]
Personal Hygiene at admission	COD_25_3	Integer	[0,1,3,4,5]
Dressing at admission	COD_25_4	Integer	[0,2,5,8,10]
Bowel control at admission	COD_25_5	Integer	[0,2,5,8,10]
Bladder control at admission	COD_25_6	Integer	[0,2,5,8,10]
Toilet at admission	COD_25_7	Integer	[0,2,5,8,10]
Chair/bed transfers at admission	COD_25_8	Integer	[0,3,8,12,15]
Ambulation at admission	COD_25_9	Integer	[0,3,8,12,15]
Stair climbing at admission	COD_25_10	Integer	[0,2,5,8,10]
Wheelchair at admission	COD_25_11	Integer	[0,1,3,4,5]
Barthel Index when discharging	COD_26	Integer	[0–100]
Feeding when discharging	COD_26_1	Integer	[0,2,5,8,10]
Bathing when discharging	COD_26_2	Integer	[0,1,3,4,5]
Personal Hygiene when discharging	COD_26_3	Integer	[0,1,3,4,5]
Dressing when discharging	COD_26_4	Integer	[0,2,5,8,10]
Bowel control when discharging	COD_26_5	Integer	[0,2,5,8,10]
Bladder control when discharging	COD_26_6	Integer	[0,2,5,8,10]
Toilet when discharging	COD_26_7	Integer	[0,2,5,8,10]
Chair/bed transfers when discharging	COD_26_8	Integer	[0,3,8,12,15]
Ambulation when discharging	COD_26_9	Integer	[0,3,8,12,15]
Stair climbing when discharging	COD_26_10	Integer	[0,2,5,8,10]
Wheelchair when discharging	COD_26_11	Integer	[0,1,3,4,5]

**Table 3:** Encoding of Social fields.

MaritalStatus	1,3,4,5,7,8,	2,6	9	LevelOfEducation	1,2,3	4,5	9
MaritalStatus_Alone	1	0	0	LevelOfEducation_high	0	1	0
MaritalStatus_NotAlone	0	1	0	LevelOfEducation_low	1	0	0

**Table 4:** Classes description for COD_1: pathology responsible for the rehabilitation intervention.

Category	Patology classes
1	Hereditary and degenerative diseases of the central nervous system
2	Other disorders of the central nervous system
3	Cerebrovascular disease
4	Diseases of the musculoskeletal system and connective tissue
5	Symptoms, signs, and ill-defined conditions
6	Fracture
7	Late effects of injuries, poisonings, toxic effects, and other external causes
8	Complications of surgical and medical care, not elsewhere classified
9	Organ or tissue replaced by other means

**Table 5:** Category description for COD_2: main pathology.

Cat.	Pathology classes for neurologic subjects	Cat.	Pathology class for orthopedic subjects
1	Stroke	8	Hip prosthesis
2	Parkinson’s disease	9	Knee prosthesis
3	Multiple sclerosis	10	Femur Internal fixation
4	Neoplasm of brain	11	Fracture of spine and trunk
5	Neurological disorders post trauma	12	Amputation
6	Diseases of spinal cord	13	Other orthopedic
7	Others neurologic

**Table 6:** Category description for COD_3-10: associated pathologies or comorbidities.

Cat.	Class of pathology	Cat.	Class of pathology
1	Others	10	Arrhythmia
2	Hematologic	11	Disorders of lipoid metabolism
3	Metabolic diseases	12	Cancer
4	Hepatic	13	Rheumatic/orthopedic
5	Neurologic	14	Circulatory complication
6	Respiratory	15	Intestinal complication
7	Diabetic	16	Kidney or urinary complication
8	Hypertension	17	Not known
9	Cardiopathic

**Table 7:** Possible values of the Manipulation impairments.

Value	description
1	no difficulties
2	difficulty in prehension of upper limb rs
3	difficulty in prehension of upper limb ls
4	difficulty in prehension of both upper limbs
5	impossibility of upper limb prehension rs
6	impossibility of upper limb prehension ls
7	impossibility of prehension in both upper limbs
999	not assessable

**Table 8:** Summary of the Datasets provided (Rows=patients; columns=Features).

File	rows	columns
Dataset.csv	3928	53
Cleaned_ Dataset.csv	3419	82
Orthopedic.csv	1844	75
Neurologial.csv	1575	76

### 1.1 Brief desciprion of the Barthel index

Since 1950, researchers started working on indices for evaluating the ADL (Activities Daily Living) of patients, which refers to patients’ daily self-care activities.

Even if several indices are available, a standard measure of it has not been approved yet. Among the various indices introduced, the Barthel Index (BI), first introduced in [5] and later revised in [8], is mostly used due to its reliability and validity, covering a wider range of conditions than any other single index. The BI is a score ranging from 0 to 100, which evaluates the patient’s condition based on ten major daily life activities. Each of the major activities is scored with 5 potential values, where the higher the value the better the ability in performing the task with zero reflecting the total inability in performing the task as reported in Table 1. Activities have different rankings and the maximal value for each category reflects the overall weighting for that activity in terms of the total score. In Table 1 the potential scores for each activity are reported.^2^

Summing up the score obtained on each activity returns the overall value of the BI where a score ranging between 0 and 20 suggests total dependence, 21–60 severe dependence, 61–90 moderate dependence, and 91–99 slight dependence. Finally, a score of 100 indicates that the patient is fully independent of assistance from others. Normally, the index is computed at the beginning of the rehabilitation treatment and at the end of it to assess the effectiveness of the rehabilitation treatment.

## 2 Experimental design, materials, and methods

Data were extracted from the Acceptance/Discharge Rehabilitation (ADR) form, which is compliant with the Italian law (DGR 731/2005). Among all the information available in the ADR form, only non-sensitive data are considered in order to make data privacy-consistent and do not allow to retrieve private information about specific patients. Data are described in the next section.

### 2.1 Raw Data

The raw dataset is stored in the spreadsheet file “Dataset.csv” and collects data of 3928 patients entering a rehabilitation program at San Raffaele hospital in Rome, Italy, between 2015 and 2018. The 53 records (features) remaining after anonymization and available in the *Raw Dataset* are reported in Table 2. For each feature (column) we report the explanatory name, the associated code reported on the first row of the spreadsheet, the type of values (integer, binary, categorical) and the range of possible values as reported on the ADR form. We remark that although encoded as integers most of the features are indeed categorical in the sense that they classify the patient on the basis of qualitative statements. As a matter of example “Marital status” allows 9 different classes coded by numbers from 1 to 9 which corresponds to possible relationship status: not married (1), married (2), separated (3), divorced (4), widowed (5) etc. Nevertheless, we prefer to report on Table 2 the kind of coding that it is found in the file rather than the interpretation.

In the Table, the notation [a−b] means that all the integer values in the range from *a* to *b* are present in the data, [*a, b*] means that only the values *a* and *b* are present, while the notation [...]+c means that in addition to the set of number specified in the squared brackets also the value *c* is present in the dataset. This last notation is used to identify missing values (coded by 99) and not assessable values (coded as 999).

The features are grouped into five main streams: demographic data, pathologies (codes 1–10), impairments (codes 11–24), Barthel index at admission (code 25 and the subcategories code25_1 to code25_11), Barthel Index at discharge (code 26 and the subcategories code26_1 to code26_11).

The pathology responsible for the rehabilitation intervention (orthopedical or neurological - CODE 1), the main pathology (CODE 2) and the associated pathologies or comorbidities (CODE 3–10) if any, are reported following the standard International Classification of Diseases, Ninth Revision, Clinical Modification (ICD9-CM) [1].

Raw data present records with inconsistencies or rare/peculiar evolution. Further there are fields not filled in (neither with 99 or 999). So we perform a cleaning and preprocessing procedure with doctors’ support which includes features encoding as well. In particular, the process was twofold along the records (eliminating patients) and the features (eliminating fields).

The availability of a cleaned and encoded dataset might be a useful data-source for applying data-driven methodologies, e.g. for predicting the discharging Barthel index, as well as setting up a standard preprocessing procedure for this kind of data.

### 2.2 Cleaned and encoded dataset

We provide the additional file “Cleaned_Dataset.csv”. As a first step, we have filtered out all those records (patients) with some inconsistencies or rare/peculiar evolution. All the following records were filtered out:

• Patients without information about Barthel Index either at the admission or at discharging;
• Patients with BI at admission less than 10 or greater than 49 because these represent exceptions in the hospitalization in a rehabilitation program, according to standard rule;
• Post-comatose and dyalitcs patients (6 and 1 patients respectively), being too pathological patients, and hence “outliers” which might compromise further statistical analysis;
• Patients whose condition deteriorated during the hospitalization (i.e. whose BI has decreased during the hospitalization period);
• Patients who have not experienced an improvement in any of the 10 components of the Barthel index;
• Patients who have been hospitalized for more than 79 days or less than 5 days.

This first cleaning procedure has deleted 510 records, leaving 3419 patients.

As a second processing phase, we operated an encoding of the values of the features. Sex, Age and Hospitalization days do not require any processing.

Concerning the feature “MaritalStatus”, which can take nine values including not known (99), the values can be divided into three categories:

• Values [2,6]: the patient is in a stable relationship
• Values [1,3,4,5,7,8]: the patient is single
• Value [99]: not known

As a consequence, the values of “Marital status” were encoded through a one-hot-encoding with two binary features: “MaritalStatus_Alone” and “MaritalStatus_NotAlone” as reported in Table 3. Note that the value [99] is implicitly encoded and so dropped from the final dataset as usually done in the One-hot-encoding procedure.

Similarly, the values of “LevelOfEducation”, which can take six values, can be divided into three categories:

• Value [1,2,3]: low level of education (less than 13 years);
• Value [4,5]: high level of education;
• Value [99]: not known.

As in the previous case, a one-hot-encoding procedure was used introducing two binary features: “Low” and “High” and the value [99] was dropped since implicitly defined by the other two, as reported in Table 3.

Concerning those features related to the patients’ diseases (code 1–10) which are encoded according to ICD9-CM, we need a more complex processing involving medical knowledge too. These features are difficult to be treated because of the large set of potential categorical values they can assume which are the codes in the ICD9-CM list, and a standard one-hot-encoding procedure would increase too much the features’ dimensionality.

Thanks to medical insight, we proceed by grouping ICD9-CM codes into classes of pathologies. Different classes have been defined according to the ADR code (1,2 or 3–10) as described in the following. The exact correspondence of each ICD9-CM code with the correct class can be found in the file “Encoding Features.xlsx”.

• **Pathology responsible for the rehabilitation intervention (code 1)**. The 4054 values of the ICD9-CM are grouped into nine categories. Hence the column corresponding to COD_1 is split into nine columns from COD_1_Cat_1 to COD_1_Cat_9. The values are encoded according to a standard one-hot-encoding procedure. In Table 4 the categories with the subcode is reported. The full correspondence of each ICD9-CM code with the correct category is available in the Sheet “COD_1” of the file “Encoding Features.xlsx”.
• **Main pathology (code 2)**. This code refers to either neurologic or orthopedic pathologies. In this case, the 4054 ICD9-CM values are grouped in 13 classes (seven for neurologic patients and six for orthopedic patients) from COD_2_Cat_1 to COD_2_Cat_13 and further encoded through a one-hot-encoding. Table 5 reports the categories, while the full table to associate each ICD9-CM code to a specific class is available in the Sheet “COD_2” of the spreadsheet file “Encoding Features.xlsx”.
• **Associated pathologies or comorbidities (code 3–10)**. These 8 features are all referring to the comorbidities of the patients. In this case we decide to proceed in a different way. First 17 categories of disease (including the field Not Known) are defined and each ICD9-CM code is assigned to a category. The 17 categories are reported in Table 6, whereas the full correspondence of each ICD9-CM code to a class is in the Sheet “COD_3-10” of the spreadsheet file “Encoding Features.xlsx”. Then, we defined a new feature for each category with name COD_3-10_cat_1 to COD_3-10_cat_17 assigned as value the number of diseases for each category.
• **Impairments (code 11 to 23)**. For each code representing a kind of impairment, the value specifies info about the kind of severity. However, there is not a relationship between the values and the level of severity, namely a large value does not indicate a more severe impairment. As a matter of example we report the list of Manipulation impairments in Table 7.
  As this is the case, we prefer to use a simple binary encoding, using 0 if the patient presents the impairment (whatever the severity) or it not assessable and 1 otherwise (not present). Note that COD_24 has been eliminated in the first cleaning phase eliminating post-comatose patients.

At the end of this procedure, we end up with 3419 rows and 82 features.

### 2.3 Neurological and orthopedic datasets

We provide two additional files “Neurological.csv” and “Orthopedic.csv”.

Indeed using the categories associated to code 2 - “Main pathology” (see Table 5), patients can be split into two main classes: those with category from 1 to 7 are from the set of “Neurological.csv” while the others are in the “Orthopedic.csv”. This might help in having more homogeneous data.

The number of neurological patients is 1575, whereas the other 1844 are orthopedic.

Of course patients classified with neurological will have all a zeros in the columns COD_2_8 to COD_2_13 (which refers to orthopedic main pathologies) that can be eliminated from the dataset because do not add any information; vice versa the orthopedic patients will have zeros in the columns from COD_2_1 to COD_2_7.

Introducing this distinction, we end up with two datasets: a dataset with neurological patients made up of 1575 patients and 76 features available in the spreadsheet file “Neurological.csv” and a dataset with orthopedic patients made up of 1844 patients and 75 features available in the spreadsheet file “Orthopedic.csv”.

A summary of the characteristic of the four datasets provided is reported in Table 8.

## Appendix A Supplementary materials

Supplementary Data S1 Supplementary Raw Research Data. This is open data under the CC BY license http://creativecommons.org/licenses/by/4.0/ Supplementary Data S1

## Footnotes

2 Please note that values for “Wheelchair” are present only in case “Ambulation” is equal to 0.
